# Incidence and Survival Outcomes of Gastrointestinal Stromal Tumors

**DOI:** 10.1001/jamanetworkopen.2024.28828

**Published:** 2024-08-19

**Authors:** Christian S. Alvarez, M. Blanca Piazuelo, Tania Fleitas-Kanonnikoff, Jennifer Ruhl, J. Alejandro Pérez-Fidalgo, M. Constanza Camargo

**Affiliations:** 1 Division of Cancer Epidemiology and Genetics, National Cancer Institute, Rockville, Maryland; 2 Department of Medicine, Vanderbilt University School of Medicine, Nashville, Tennessee; 3 Department of Medical Oncology, Instituto de Investigación Sanitaria (INCLIVA) Biomedical Research Institute, University of Valencia, Valencia, Spain; 4 Division of Cancer Control and Population Sciences, National Cancer Institute, Rockville, Maryland; 5 Medical Oncology Department, University Hospital of Valencia, INCLIVA Biomedical Research Institute, Valencia, Spain; 6 Centro de Investigación Biomédica en Red Cáncer, Valencia, Spain

## Abstract

**Importance:**

The incidence of gastrointestinal stromal tumors (GISTs) increased after the implementation of GIST-specific histology coding in 2001, but updated data on trends and survival are lacking.

**Objective:**

To examine the evolving epidemiology of GISTs in major organ sites.

**Design, Setting, and Participants:**

This descriptive, population-based cohort study used nationally representative data from the National Cancer Institute Surveillance, Epidemiology, and End Results (SEER) Program, including the SEER-22 and SEER-17 registries. Data were from evaluated patients aged 20 years or older with GISTs diagnosed between January 1, 2000, and December 31, 2019. Analyses were last updated on October 29, 2023.

**Main Outcomes and Measures:**

Organ site-specific trends in age-standardized incidence rates and annual percent changes (APCs) in rates were estimated by race and ethnicity and, when possible, by sex, age, and primary indicator. Multivariable Cox proportional hazards regression models were used to examine racial and ethnic differences in overall and GIST-specific survival by site.

**Results:**

The SEER-22 and SEER-17 datasets contained 23 001 and 12 109 case patients with GISTs, respectively. Patients in the SEER-22 registry had a mean (SD) age of 64 (13) years and 51.3% were men. With regard to race and ethnicity, 9.7% of patients were Asian or Pacific Islander, 12.3% were Hispanic, 19.6% were non-Hispanic Black, and 57.7% were non-Hispanic White. Overall incidence rates of GISTs in the SEER-22 cohort increased substantially over time for all organ sites but the colon (APCs: esophagus, 7.3% [95% CI, 4.4% to 10.2%]; gastric, 5.1% [95% CI, 4.2% to 6.1%]; small intestine, 2.7% [95% CI, 1.8% to 3.7%]; colon, −0.2% [95% CI, −1.3% to 0.9%]; and rectum, 1.9% [95% CI, 0.1% to 3.8%]). There were similar increasing trends by age groups (<50 vs ≥50 years), sex, race and ethnicity, and primary indicator for gastric and small intestine GISTs. Increases were mainly restricted to localized stage disease. Patients in the SEER-17 cohort had a mean (SD) age of 64 (14) years and 51.9% were men. With regard to race and ethnicity, 13.3% of patients were Asian or Pacific Islander, 11.6% were Hispanic, 17.8% were non-Hispanic Black, and 56.6% were non-Hispanic White. Non-Hispanic Black individuals had higher overall mortality for esophageal (adjusted hazard ratio [HR], 6.4 [95% CI, 2.0 to 20.3]) and gastric (adjusted HR, 1.4 [95% CI, 1.2 to 1.5]) GISTs compared with non-Hispanic White individuals. Asian or Pacific Islander individuals also had higher overall mortality for esophageal GISTs (adjusted HR, 5.6 [95% CI, 1.5 to 20.2]). Results were similar for GIST-specific survival.

**Conclusions and Relevance:**

In this cohort study using SEER data, the incidence of GISTs in major organ sites increased in the last 2 decades among several population groups. These findings suggest that additional studies are warranted to identify risk factors, because histologic reclassification and higher availability of endoscopy and imaging do not fully explain these unfavorable incidence trends. Prevention efforts are needed to reduce the substantial survival disparities among racial and ethnic minoritized populations.

## Key Points

**Question:**

Has the epidemiology of gastrointestinal stromal tumors (GISTs) changed in recent decades?

**Findings:**

In this cohort study of 23 001 patients, age-adjusted incidence rates for common digestive GISTs increased between 2% and 7% during 2000 and 2019, mostly for early-stage tumors. Survival disparities among racial and ethnic groups were found for some organ sites.

**Meaning:**

These findings suggest that the incidence of GISTs in major organ sites has increased in the last 2 decades among several population groups and may help to prioritize future research directions to reduce the burden of disparities of GISTs in the US.

## Teaser

This cohort study uses National Cancer Institute Surveillance, Epidemiology, and End Results registry data to investigate the evolving epidemiology gastrointestinal stromal tumors in major organ sites.

## Introduction

Gastrointestinal stromal tumors (GISTs) are the most common mesenchymal tumors of the gastrointestinal tract. Gastrointestinal stromal tumors originate primarily from the interstitial cells of Cajal or their precursors.^1,2^ Most GISTs develop in the stomach and small intestine, and they typically occur in older adults, with a similar sex distribution.^3,4,5^

Gastrointestinal stromal tumors were recognized as a distinct tumor type in 1998, when Hirota et al^6^ identified gain-of-function mutations in *KIT* as a key oncogenic driver. Most GISTs are associated with activating mutations in either *KIT* or *PDGFRA*.^6,7,8^ Before the use of *KIT* (CD117) immunostaining as a GIST marker in the early 2000s,^9^ most GISTs were diagnosed as smooth muscle tumors (eg, leiomyosarcomas), neurofibromas, or schwannomas.^3,10,11^

The behavior of GISTs ranges from incidental small tumors to those with extensive metastasis. The risk of malignant behavior is based on anatomic location, tumor size, and mitotic activity.^9,10,12,13,14^ Survival rates of GISTs greatly improved after 2002, following the introduction of the tyrosine kinase inhibitor (TKI) imatinib mesylate.^15,16,17^

A sharp increase in the incidence of GISTs was observed in 2000 to 2005, mainly due to reclassification of sarcomas as GISTs and increased disease awareness.^15,16,18^ Until recently, most GISTs with favorable characteristics had been classified as “benign” tumors or tumors “with uncertain malignant potential” (using the first update of the *International Classification of Diseases for Oncology, Third Edition* [*ICD-O-3.1*], released in 2013) and, therefore, were not recorded.^11,19^ The second and most recent update, *ICD-O-3.2* (released in 2019), classifies all GISTs as malignant; therefore, an increase in incidence after 2020 is expected. In this study, we evaluated recent GIST incidence trends and survival outcomes by race and ethnicity using data from the National Cancer Institute Surveillance, Epidemiology, and End Results (SEER) Program.

## Methods

The National Cancer Institute deemed this cohort study to be exempt from institutional review board approval because publicly available deidentified data were used. Thus, informed consent was not required. The study followed the Strengthening the Reporting of Observational Studies in Epidemiology (STROBE) reporting guideline.

### Data Source and Case Selection

Our analyses were based on data for case patients in the SEER-22 and SEER-17 databases (January 1, 2000, through December 31, 2019). We used histologic code 8936/3 to identify patients with primary and nonprimary GISTs for the following organ sites: esophagus (C15), gastric (C16.0 [cardia], C16.1-6 [noncardia], and C16.8-9 [overlapping or unspecified]), small intestine (C17), colon (C18 and C26.0), and rectum (C19.9 and C20.9). We excluded individuals aged younger than 20 years given the low number (n = 80 for SEER-22 and n = 53 for SEER-17). We also excluded cases that originated from other organs (n = 2632 in SEER-22 and n = 1453 in SEER-17 [45% represented other digestive organs; 17% soft tissues; 21% peritoneum, retroperitoneum, omentum, and mesentery; and 17% miscellaneous or other organs]). Because most tumors referred to as leiomyosarcomas in the older literature are GISTs, we also evaluated leiomyosarcomas (*ICD-O-3* code 8890/3) for the same organs for comparison. We used (original or combined) predefined SEER*Stat variables. We did not examine the data for 2020 because it was an anomalous year caused by the COVID-19 pandemic with consequential delays in screening and diagnosis of GISTs and other tumors.

We examined racial and ethnic differences in overall and GIST-specific survival by site due to persistent disparities in access to health care experienced by racial and ethnic marginalized populations. The definitions of race and ethnicity follow the criteria established by the SEER Program^20^ and are presented as American Indian or Alaska Native, Asian or Pacific Islander, Hispanic, non-Hispanic Black (hereinafter Black), non-Hispanic White (hereinafter White), or unknown.

### Statistical Analysis

Using SEER-22 data, we calculated age-standardized incidence rates (ASRs) and annual percentage changes (APCs) with SEER*Stat, version 8.4.1 (National Cancer Institute), and Joinpoint, version 4.8.0.1 (National Cancer Institute). Using SEER-17 data, we calculated median overall survival and cancer-specific survival as well as 5-year relative survival rates using Stata, version 18.0 (StataCorp LLC). Multivariable Cox proportional hazards regression models were used to compute hazard ratios (HRs) for overall and GIST-specific mortality. Statistical significance was set at *P* < .05 (2-tailed), and analyses were last updated on October 29, 2023.

## Results

The SEER-22 and SEER-17 datasets contained 23 001 and 12 109 case patients with GISTs, respectively. The SEER-22 cohort had a mean (SD) age of 64 (13) years; 51.3% of patients were men and 48.7% were women. A total of 0.2% of patients were American Indian or Alaska Native, 9.7% were Asian or Pacific Islander, 19.6% were Black, 12.3% were Hispanic, and 57.7% were White; race and ethnicity was unknown for 0.5%. The SEER-17 cohort had a mean (SD) age of 64 (14) years; 51.9% of patients were men and 5822 (48.1%) were women. A total of 0.2% of patients were American Indian or Alaska Native, 13.3% were Asian or Pacific Islander, 17.8% were Black, 11.6% were Hispanic, and 56.6% were White; race and ethnicity was unknown for 0.5%. In the SEER-17 cohort, 9931 patients (82.0%) had primary tumors, 6264 (51.7%) had localized disease, and 9772 (80.7%) were treated surgically.

### Esophageal GIST

**Figure 1.**
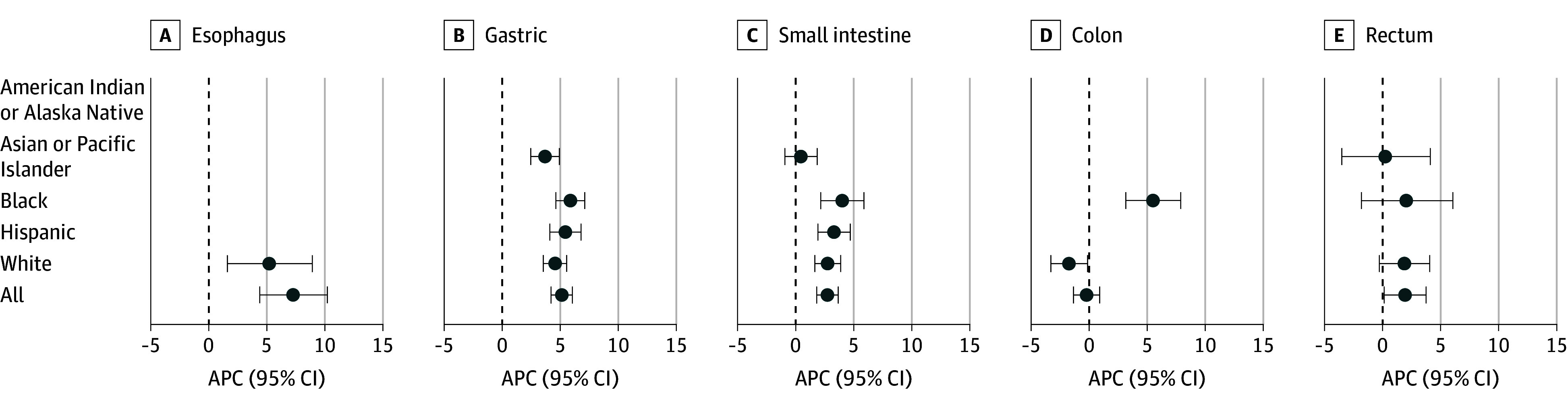
Race and Ethnicity–Specific Average Annual Percentage Changes (APCs) for Gastrointestinal Stromal Tumors by Organ Site Data are from the National Cancer Institute Surveillance, Epidemiology, and End Results Program SEER-22 registry for 2000 to 2019. Missing APCs could not be calculated due to limited case counts.

Of the 159 patients (93 men [58.5%] and 66 women [41.5%]) in the SEER-22 cohort with esophageal GISTs, 122 (76.7%) had primary tumors (eTable 1 in Supplement 1). Five patients or fewer (0.6%) were American Indian or Alaska Native, 11 (6.9%) were Asian or Pacific Islander, 28 (17.6%) were Black, 19 (12.0%) were Hispanic, and 99 (62.3%) were White; race and ethnicity was unknown for up to 5 patients (0.6%). The overall ASR of esophageal GIST was 0.005 per 100 000 persons in 2000 and increased to 0.014 per 100 000 persons by 2019, with an APC of 7.3% (95% CI, 4.4% to 10.2%). The overall ASR of esophageal leiomyosarcoma was 0.005 per 100 000 persons in 2000 and decreased to 0 per 100 000 persons by 2019. The APC for both tumor types combined was 4.1% (95% CI, 1.5% to 6.6%). eFigure 1 in Supplement 2 displays the 5-year incidence rates for GIST and leiomyosarcomas. The APC for White individuals was 5.21 (Figure 1).

**Figure 2.**
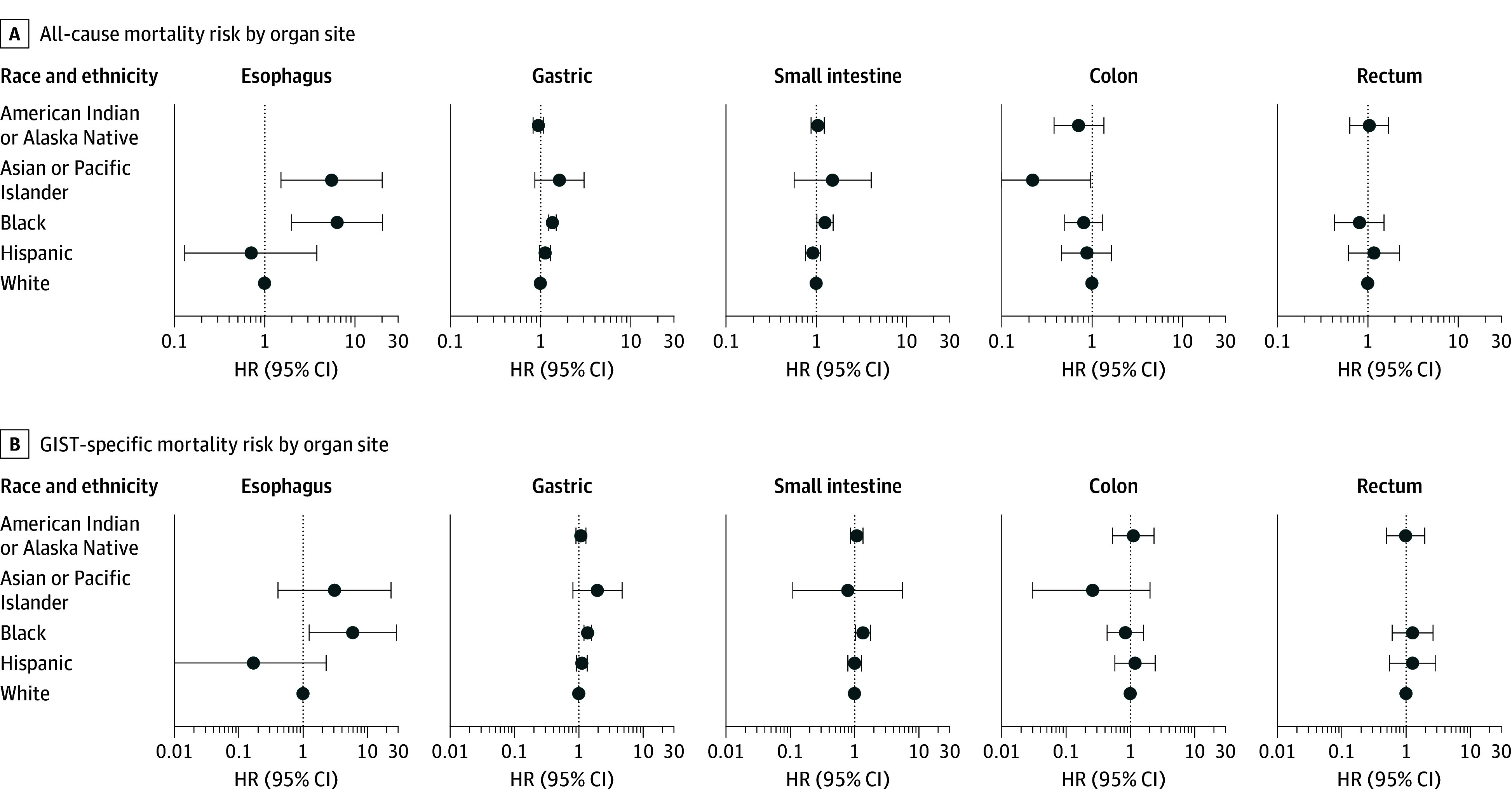
Race and Ethnicity–Specific Adjusted Hazard Ratios (HRs) of All-Cause and Gastrointestinal Stromal Tumor (GIST)-Specific Mortality Risk by Organ Site A and B, All-cause (A) and GIST-specific (B) mortality risk by organ site, using data from the National Cancer Institute Surveillance, Epidemiology, and End Results Program SEER-17 registry for 2000 to 2019. Hazard ratios were adjusted for age, sex, year of diagnosis, pathologic grade (ie, mitotic rate), clinical stage, primary indicator, surgical treatment, and income. Gastric GIST models were also adjusted for anatomic subsite.

Of the 84 patients (52 men [61.9%] and 32 women [38.1%]) in the SEER-17 cohort with esophageal GISTs, 26 (31.0%) had primary tumors (Table 1). A total of 8 patients (9.5 %) were Asian or Pacific Islander, 13 (15.5%) were Black, 8 (9.5%) were Hispanic, and 54 (64.3%) were White; no patients were American Indian or Alaska Native, and race and ethnicity was unknown for up to 5 patients (1.1%). The median overall survival for Asian or Pacific Islander, Black, Hispanic, and White individuals was 8.8, 3.6, 10.3, and 15.3 years, respectively; Black individuals had the lowest survival rate. The multivariable Cox proportional hazards regression analysis showed that Asian or Pacific Islander patients (HR, 5.6 [95% CI, 1.5 to 20.2]) and Black patients (HR, 6.4 [95% CI, 2.0 to 20.3]) had the highest overall mortality compared with White individuals (Figure 2A). Overall survival was worse for individuals with undifferentiated or poorly differentiated (ie, with high mitotic rate) tumors (HR, 6.3 [95% CI, 1.2 to 34.7]) than for those with other or unspecified tumors after adjustment for other covariates. The GIST-specific estimates were attenuated in magnitude (Figure 2B), but the excess mortality among Black individuals (compared with White individuals) remained statistically significant.

**Table 1.** Selected Characteristics of Patients Aged 20 Years or Older Diagnosed With GISTs, SEER-17 Registry, 2000 to 2019^a^

Characteristic	Organ site (n = 12 109)				
	Esophagus (n = 84)	Gastric (n = 7799)	Small intestine (n = 3560)	Colon (n = 312)	Rectum (n = 354)
Age, y					
Mean (SD)	67 (13)	65 (13)	61 (14)	65 (13)	62 (13)
20-49	10 (11.9)	1065 (13.7)	745 (20.9)	45 (14.4)	65 (18.4)
≥50	74 (88.1)	6734 (86.3)	2815 (79.1)	267 (85.6)	289 (81.6)
Sex					
Female	32 (38.1)	3894 (49.9)	1614 (45.3)	144 (46.2)	138 (39.0)
Male	52 (61.9)	3905 (50.1)	1946 (54.7)	168 (53.8)	216 (61.0)
Race and ethnicity					
American Indian or Alaska Native	0	19 (0.2)	8 (0.2)	≤5 (1.3)	0
Asian or Pacific Islander	8 (9.5)	1036 (13.3)	460 (12.9)	25 (8.0)	85 (24.0)
Black	13 (15.5)	1746 (22.4)	277 (7.8)	68 (21.8)	49 (13.8)
Hispanic	8 (9.5)	857 (11.0)	471 (13.2)	28 (9.0)	39 (11.0)
White	54 (64.3)	4103 (52.6)	2329 (65.4)	186 (59.6)	180 (50.9)
Unknown	≤5 (1.1)	38 (0.5)	15 (0.4)	≤5 (0.3)	≤5 (0.3)
Year of diagnosis					
2000-2004	11 (13.1)	1045 (13.4)	664 (18.6)	73 (23.4)	64 (18.1)
2005-2009	15 (17.9)	1560 (20.0)	744 (20.9)	71 (22.8)	72 (20.3)
2010-2014	21 (25.0)	2329 (29.9)	1006 (28.3)	81 (26.0)	105 (30.0)
2015-2019	37 (44.0)	2865 (36.7)	1146 (32.2)	87 (27.9)	113 (32.0)
Clinical stage					
Localized	37 (44.1)	4279 (54.9)	1640 (46.1)	127 (40.7)	181 (51.1)
Regional	13 (15.5)	739 (9.5)	563 (15.8)	49 (15.7)	60 (17.0)
Distant	6 (7.1)	1069 (13.7)	662 (18.6)	59 (18.9)	33 (9.3)
Unspecified	28 (33.3)	1712 (21.9)	695 (19.5)	77 (24.7)	80 (22.6)
First malignant primary					
No	26 (31.0)	1419 (18.2)	611 (17.2)	69 (22.1)	53 (15.0)
Yes	58 (69.0)	6380 (81.8)	2949 (82.8)	243 (77.9)	301 (85.0)
Anatomic subsite					
Cardia	NA	539 (6.9)	NA	NA	NA
Noncardia	NA	4412 (56.6)	NA	NA	NA
Overlapping or unspecified	NA	2848 (36.5)	NA	NA	NA
Tumor size, mm^b^					
1-50	34 (40.5)	3277 (42.0)	1170 (32.9)	111 (35.6)	145 (41.0)
51-100	26 (30.9)	2093 (26.9)	1207 (33.9)	71 (22.8)	121 (34.2)
>100	10 (11.9)	1415 (18.1)	682 (19.1)	40 (12.8)	34 (9.6)
Unspecified	14 (16.7)	1014 (13.0)	501 (14.1)	90 (28.8)	54 (12.2)
Pathologic grade (mitotic count)^c^					
Undifferentiated or poorly differentiated^d^	≤5 (6.0)	742 (9.5)	445 (12.5)	57 (18.3)	34 (9.6)
Other or unspecified	79 (94.0)	7057 (90.5)	3115 (87.5)	255 (81.7)	320 (90.4)
Surgery					
Not performed	37 (44.0)	1612 (20.7)	398 (11.2)	64 (20.5)	89 (25.1)
Performed	47 (56.0)	6086 (78.0)	3140 (88.2)	246 (78.9)	253 (71.5)
Unspecified	0	101 (1.3)	22 (0.6)	≤5 (0.6)	12 (3.4)
Vital status					
Alive	51 (60.7)	5061 (64.9)	2198 (61.7)	159 (51.0)	233 (65.8)
Dead due to GIST	19 (22.6)	1343 (17.2)	755 (21.2)	88 (28.2)	69 (19.5)
Dead due to other causes	14 (16.7)	1395 (17.9)	607 (17.1)	65 (20.8)	52 (14.7)
Median household income (inflation-adjusted to 2019), $					
<49 999	14 (16.7)	953 (12.2)	402 (11.3)	42 (13.5)	39 (11.0)
50 000-59 999	9 (10.7)	1124 (14.4)	485 (13.6)	46 (14.7)	49 (13.8)
60 000-69 999	23 (27.4)	2357 (30.2)	1053 (29.6)	91 (29.2)	112 (31.6)
≥70 000	38 (45.2)	3365 (43.2)	1619 (45.4)	133 (42.6)	154 (43.5)
Unspecified	0	0	≤5 (0.1)	0	0
County type					
Metropolitan areas					
>1 million population	50 (59.5)	4807 (61.6)	2117 (59.4)	192 (61.5)	223 (63.0)
250 000-1 million population	19 (22.6)	1736 (22.3)	771 (21.7)	63 (20.2)	80 (22.6)
<250 000 population	8 (9.5)	516 (6.6)	263 (7.4)	24 (7.7)	20 (5.7)
Nonmetropolitan					
Adjacent to a metropolitan area	≤5 (2.4)	462 (5.9)	243 (6.8)	21 (6.7)	19 (5.4)
Not adjacent to a metropolitan area	≤5 (6.0)	273 (3.5)	163 (4.6)	12 (3.4)	12 (3.4)
Unspecified	0	≤5 (0.1)	≤5 (0.1)	0	0

Abbreviations: GIST, gastrointestinal stromal tumor; NA, not applicable; SEER, National Cancer Institute Surveillance, Epidemiology, and End Results Program.

^a^ Unless indicated otherwise, values are presented as No. (%) of patients.

^b^ Tumor size used combined predefined SEER variables (EOD 10 tumor size [before 2003], CS tumor size [2004-2015], and tumor size summary 2016); data provided by SEER upon request.

^c^ Pathologic grade used combined predefined SEER variables (grade through 2017 and pathologic grade 2018-2019).

^d^ Includes high mitotic count (>5 mitosis/5 mm^2^).

### Gastric GIST

Of the 15 085 patients (7465 men [49.5%] and 7620 women [50.5%]) in the SEER-22 cohort diagnosed with gastric GIST, 12 426 (82.4%) had primary tumors (eTable 1 in Supplement 1). A total of 32 patients (0.2%) were American Indian or Alaska Native, 1427 (9.5%) were Asian or Pacific Islander, 3681 (24.4%) were Black, 1780 (11.8%) were Hispanic, and 8088 (53.6%) were White; race and ethnicity was unknown for 77 patients (0.5%). The overall ASR of gastric GIST was 0.24 per 100 000 persons in 2000 and increased to 0.93 per 100 000 persons by 2019, with an APC of 5.1% (95% CI, 4.2% to 6.1%). In contrast, the overall ASR of gastric leiomyosarcoma was 0.075 per 100 000 persons in 2000 and decreased to 0.009 per 100 000 by 2019, with a corresponding APC of −10.2% (95% CI, −13.3% to −7.0%). The APC for both tumor types combined was 4.8% (95% CI, 4.0% to 5.5%). Substantial increases in GIST incidence were observed for all anatomic subsites, with APCs of 3.5% (95% CI, 2.0% to 5.0%) for cardia subsites, 6.2% (95% CI, 5.2% to 7.2%) for noncardia subsites, and 3.9% (95% CI, 2.8% to 5.0%) for overlapping or unspecified tumors.

**Figure 3.**
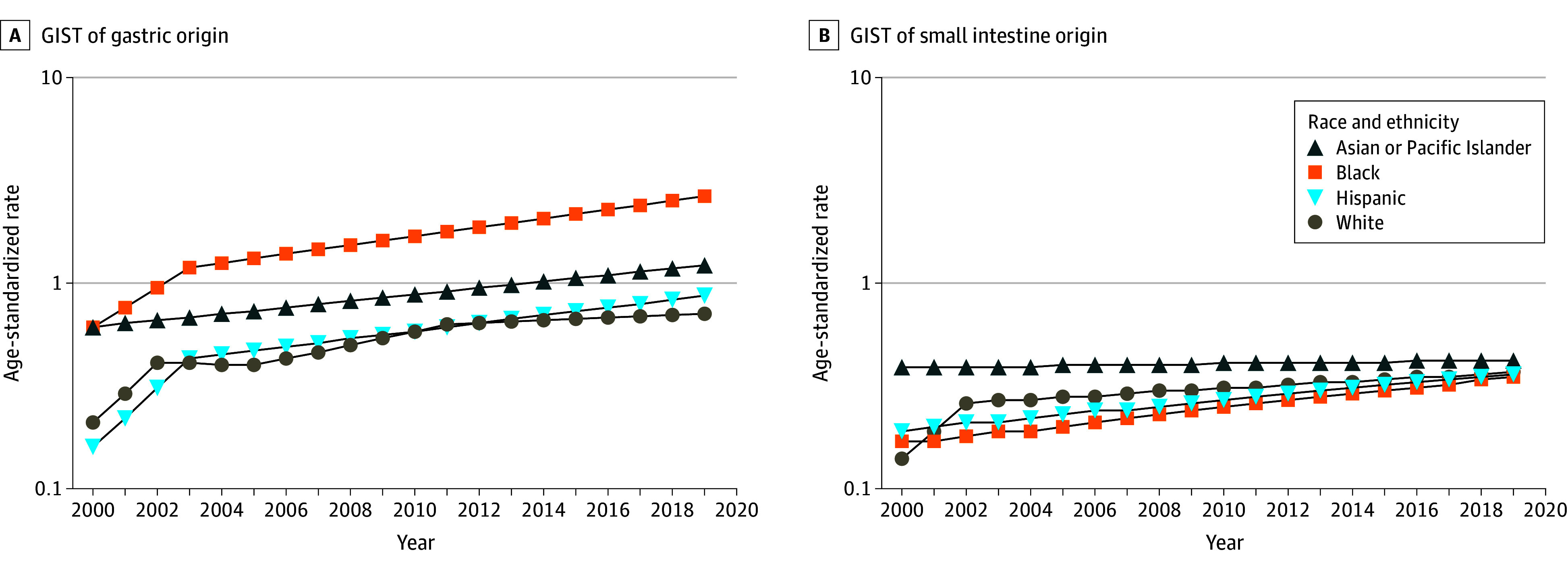
Race and Ethnicity–Specific Age-Standardized Incidence Trends for Gastrointestinal Stromal Tumors (GISTs) of Gastric and Small Intestine Origin A and B, Gastrointestinal stromal tumors of gastric (A) and small intestine (B) origin, using data from the National Cancer Institute Surveillance, Epidemiology, and End Results Program SEER-22 registry for 2000 to 2019.

Race and ethnicity–specific trends are presented in Figure 3A. Asian or Pacific Islander individuals and Black individuals had the highest rates of gastric GISTs. Incidence trends increased across all racial and ethnic groups (Figure 1), with APCs ranging from 3.7% (Asian or Pacific Islander) to 5.9% (Black). The increased incidence occurred in both sexes and across all strata of age, primary indicator, and median household income (eTable 2 in Supplement 1). Rates of localized tumors increased substantially for all racial and ethnic groups (Table 2). Notably, rates of distant tumors substantially increased in Black individuals and all individuals combined.

**Table 2.** Clinical Stage-Specific APCs in Incidence of GISTs Across Racial and Ethnic Groups, SEER-22 Registry, 2004 to 2017^a^

Stage	Overall, APC (95% CI)	Race and ethnicity, APC (95% CI)			
		Asian or Pacific Islander	Black	Hispanic	White
Gastric					
Localized	8.1 (6.4 to 9.9)	7.0 (3.9 to 10.2)	9.7 (7.5 to 11.9)	8.7 (6.0 to 11.5)	7.3 (5.4 to 9.2)
Regional	−0.9 (−3.0 to 1.3)	−3.1 (−8.7 to 2.8)	0.6 (−3.9 to 5.4)	−5.2 (−8.9 to −1.3)	−2.1 (−4.9 to 0.7)
Distant	2.7 (1.1 to 4.3)	0.2 (−3.5 to 4.1)	3.6 (0.5 to 6.9)	2.9 (−1.5 to 7.5)	1.9 (−0.1 to 4.0)
Unspecified	−2.9 (−5.0 to −0.7)	−4.5 (−9.1 to 0.4)	−3.3 (−6.6 to 0.1)	−2.0 (−7.5 to 3.8)	−3.0 (−6.0 to 0.1)
Small intestine					
Localized	5.2 (3.8 to 6.6)	4.7 (2.1 to 7.3)	6.4 (2.8 to 10.1)	8.1 (5.0 to 11.2)	4.3 (2.7 to 6.0)
Regional	−0.6 (−2.9 to 1.7)	−1.2 (−6.8 to 4.7)	−6.3 (−11.4 to −0.9)	0.2 (−3.5 to 4.0)	−0.5 (−3.2 to 2.3)
Distant	1.0 (−0.9 to 2.9)	−3.1 (−8.4 to 2.4)	NA	1.6 (−1.3 to 4.5)	1.7 (−0.7 to 4.1)
Unspecified	−6.7 (−10.0 to −3.3)	−4.9 (−10.8 to 1.3)	NA	NA	−7.5 (−12.1 to −2.8)
Colon					
Localized	4.6 (2.0 to 7.2)	NA	8.2 (3.9 to 12.7)	NA	5.2 (0.4 to 10.3)
Regional	−4.5 (−9.7 to 1.1)	NA	NA	NA	−4.9 (−12.5 to 3.2)
Distant	−3.9 (−9.8 to 2.4)	NA	NA	NA	−4.8 (−12.7 to 3.7)
Unspecified	−7.7 (−12.9 to −2.3)	NA	NA	NA	−6.4 (−11.8 to −0.7)
Rectal					
Localized	7.6 (3.1 to 12.4)	NA	9.0 (0.5 to 18.2)	NA	5.9 (1.5 to 10.5)
Regional	2.0 (−3.8 to 8.3)	NA	NA	NA	2.1 (−5.7 to 10.4)
Distant	7.7 (0.4 to 15.5)	NA	NA	NA	NA
Unspecified	−8.7 (−15.0 to −1.8)	NA	NA	NA	NA

Abbreviations: APC, annual percentage change; GIST, gastrointestinal stromal tumor; NA, not applicable; SEER, National Cancer Institute Surveillance, Epidemiology, and End Results Program.

^a^ Limited case counts prevented calculation of APCs for esophageal GIST for all groups, and all organs for American Indian or Alaska Native individuals. Clinical stage data before 2004 and after 2017 were not included because all cases were classified as unspecified.

Of the 7799 patients (3905 men [50.1%] and 3894 women [49.9%]) in the SEER-17 cohort diagnosed with gastric GIST, 6380 (81.8%) had primary tumors and 4412 (56.6%) had tumors localized to noncardia subsites (Table 1). A total of 19 patients (0.2%) were American Indian or Alaska Native, 1036 (13.3%) were Asian or Pacific Islander, 1746 (22.4%) were Black, 857 (11.0%) were Hispanic, and 4103 (52.6%) were White; race and ethnicity was unknown for 38 patients (0.5%). The median survival for American Indian or Alaska Native, Asian or Pacific Islander, Black, Hispanic, and White individuals was 8.5, 13.7, 9.1, 12.6, and 11.8 years, respectively; American Indian or Alaska Native individuals and Black individuals had the lowest survival rates. The multivariable Cox proportional hazards regression analysis showed that American Indian or Alaska Native individuals (HR, 1.6 [95% CI, 0.9 to 3.0]) and Black individuals (HR, 1.4 [95% CI, 1.2 to 1.5]) had the highest mortality rates compared with White individuals (Figure 2A). Older age, male sex, poorly differentiated histology, and distant stage were all associated with worse survival. In contrast, primary indicator, higher income, and recent year of diagnosis were associated with better survival. Tumor location (cardia vs noncardia) was not associated with survival. Gastrointestinal stromal tumor–specific mortality results were similar (Figure 2B). Over the 20-year study period, the 5-year relative survival for patients with regional and distant disease combined ranged from 24.0% in American Indian or Alaska Native individuals to 62.0% in Asian or Pacific Islander individuals (eFigure 2 in Supplement 2).

### Small Intestine GIST

Of the 6502 patients (3526 men [54.2%] and 2976 women [45.8%]) in the SEER-22 cohort diagnosed with small intestine GIST, 5439 (83.7%) had primary tumors (eTable 1 in Supplement 1). A total of 18 patients (0.3%) were American Indian or Alaska Native, 645 (9.9%) were Asian or Pacific Islander, 547 (8.4%) were Black, 907 (14.0%) were Hispanic, and 4362 (67.1%) were White; race and ethnicity was unknown for 23 patients (0.3%). The overall ASR of small intestine GISTs was 0.13 per 100 000 persons in 2000 and increased to 0.36 per 100 000 persons by 2019, with an APC of 2.7% (95% CI, 1.8% to 3.7%). In contrast, the overall ASR of leiomyosarcoma of the small intestine was 0.06 per 100 000 persons in 2000 and decreased to 0.01 per 100 000 persons by 2019, with an APC of −5.8% (95% CI, −8.8% to −2.7%). The APC for both tumor types combined was 2.3% (95% CI, 1.6% to 3.0%). eFigure 1 in Supplement 2 displays the 5-year incidence rates for GIST and leiomyosarcomas of the small intestine. Race and ethnicity–specific trends are presented in Figure 3B; Asian or Pacific Islander individuals had the highest rates. Incidence trends increased substantially across all racial and ethnic groups (Figure 1) except for Asian or Pacific Islander individuals. The increased incidence occurred in both sexes and across all strata of age, primary indicator, and median household income (eTable 2 in Supplement 1). Rates of localized tumors increased substantially for each racial and ethnic group. In contrast, rates of regional and distant staged disease decreased or plateau (Table 2).

Of the 3560 patients (1946 men [54.7%] and 1614 women [45.3%]) in the SEER-17 cohort diagnosed with small intestine GIST, 2949 (82.8%) had primary tumors (Table 1). A total of 8 patients (0.2%) were American Indian or Alaska Native, 460 (12.9%) were Asian or Pacific Islander, 277 (7.8%) were Black, 471 (13.2%) were Hispanic, and 2329 (65.4%) were White; race and ethnicity was unknown for 15 patients (0.4%). The median survival of American Indian or Alaska Native, Asian or Pacific Islander, Black, and White individuals was 6.2, 11.4, 10.2, and 9.9 years, respectively. American Indian or Alaska Native patients had the lowest survival rate. All Hispanic individuals were alive during the study period. The multivariable Cox proportional hazards regression analysis showed that Black individuals (HR, 1.3 [95% CI, 1.0-1.5]) and Asian or Pacific Islander individuals (HR, 1.5 [95% CI, 0.6-4.1]) had the highest mortality as compared to White individuals (Figure 2A). Older age, male sex, poorly differentiated histology, and distant stage were all associated with worse survival. In contrast, primary indicator, surgical treatment, higher income, and recent year of diagnosis were associated with better survival. Patients treated surgically had a 50.0% lower mortality risk (HR, 0.5 [95% CI, 0.4-0.6]) compared with those treated with other treatment modalities. Except for Asian or Pacific Islander individuals, the GIST-specific estimates were similar in magnitude (Figure 2B), and the excess mortality in Black individuals (compared with White individuals) remained statistically significant. Over the 20-year study period, the 5-year relative survival for patients with regional and distant disease combined ranged from 0% in American Indian or Alaska Native individuals to 73.3% in White individuals (eFigure 2 in Supplement 2).

### Colon GIST

Of the 577 patients (320 men [55.5%] and 257 women [44.5%]) in the SEER-22 cohort diagnosed with colon GIST, 457 (79.2%) had primary tumors (eTable 1 in Supplement 1). A total of 6 patients (1.0%) were American Indian or Alaska Native, 31 (5.4%) were Asian or Pacific Islander, 142 (24.6%) were Black, 61 (10.6%) were Hispanic, and 336 (58.2%) were White; race and ethnicity was unknown for up to 5 patients (0.2%). The overall ASR of colon GIST was 0.025 per 100 000 persons in 2000 and increased to 0.028 per 100 000 persons by 2019, with an APC of −0.2% (95% CI, −1.3% to 0.9%). The overall ASR of leiomyosarcoma of the colon was 0.014 per 100 000 persons in 2000 and decreased to 0.009 per 100 000 persons by 2019, with a corresponding APC of −1.5% (95% CI, −3.4% to 0.6%). The APC for both tumor types combined was −0.7% (95% CI, −1.6% to 0.2%). Contrasting trends were observed between Black individuals and White individuals (Figure 1), with notable increases for Black individuals and decreases for White individuals.

Of the 312 patients (168 men [53.8%] and 144 women [46.2%]) in the SEER-17 cohort diagnosed with colon GIST, 243 (77.9%) had primary tumors (Table 1). Five patients or fewer (1.3%) were American Indian or Alaska Native, 25 (8.0%) were Asian or Pacific Islander, 68 (21.8%) were Black, 28 (9.0%) were Hispanic, and 186 (59.6%) were White; race and ethnicity was unknown for up to 5 patients (0.3%). The median survival for Asian or Pacific Islander, Black, Hispanic, and White individuals was 5.4, 19.5, 11.3, and 6.3 years, respectively; Asian or Pacific Islander individuals had the lowest survival rate. The multivariable Cox proportional hazards regression analysis showed that except for Asian or Pacific Islander individuals, all racial and ethnic groups had comparable survival (Figure 2A). Older age, poorly differentiated histology, and distant stage were associated with worse survival. In contrast, surgical treatment and recent year of diagnosis were associated with better survival. Patients treated surgically had a 40.0% lower mortality risk (HR, 0.6 [95% CI, 0.4-0.9]) compared with those treated with other therapy modalities. Gastrointestinal stromal tumor–specific mortality was comparable across all racial and ethnic groups (Figure 2B).

### Rectal GIST

Of the 678 patients (405 men [59.7%] and 273 women [40.3%]) in the SEER-22 cohort diagnosed with rectal GIST, 583 (86.0%) had primary tumors (eTable 1 in Supplement 2). Five patients or fewer (0.2%) were American Indian or Alaska Native, 115 (17.0%) were Asian or Pacific Islander, 104 (15.3%) were Black, 73 (10.8%) were Hispanic, and 382 (56.3%) were White; race and ethnicity was unknown for up to 5 patients (0.4%). The ASR of rectal GIST was 0.015 per 100 000 persons in 2000 and increased to 0.033 per 100 000 persons by 2019, with a corresponding APC of 1.9% (95% CI, 0.1% to 3.8%). The ASR of rectal leiomyosarcoma was 0.002 per 100 000 persons in 2000 and 0.006 per 100 000 in 2019, with a corresponding APC of −0.2% (95% CI, −3.4% to 3.1%). The APC for both tumor types combined was 1.7% (95% CI, −0.04% to 3.5%). Race and ethnicity–specific APCs are presented in Figure 1; all groups exhibited nonsignificant increases in incidence.

Of the 354 patients (216 men [61.0%] and 138 women [39.0%]) in the SEER-17 cohort diagnosed with rectal GIST, 301 (85.0%) had primary tumors (Table 1). A total of 85 patients (24.0%) were Asian or Pacific Islander, 49 (13.8%) were Black, 39 (11.0%) were Hispanic, and 180 (50.9%) were White; no patients were American Indian or Alaska Native, and race and ethnicity was unknown for up to 5 patients (0.3%). Median survival was 15.7, 13.5, 13.8, and 11.9 years for Asian or Pacific Islander, Black, Hispanic, and White individuals, respectively; White individuals had the lowest survival rate. The multivariable Cox proportional hazards regression analysis showed that all racial and ethnic groups had comparable survival (Figure 2A). Older age and poorly differentiated histology were associated with worse survival. In contrast, surgical treatment and recent year of diagnosis were associated with better survival. Patients treated surgically had a 50.0% lower mortality risk (HR, 0.5 [95% CI, 0.3 to 0.8]) compared with those treated with other therapies. Gastrointestinal stromal tumor–specific mortality results were similar (Figure 2B).

## Discussion

In this study, we evaluated the incidence and survival of GISTs for 2000 to 2019 using data from the SEER Program. Diagnostic and nosological practice patterns affect GIST incidence trends. As expected, there was a sharp increase in incidence rates between 2000 and 2005, mainly related to coding reclassification. However, we observed a continued increase in the incidence of GISTs after 2005. Although the decreased trends of leiomyosarcoma had a complementary pattern to the increased trends of GIST for some organs, the combined burden of these tumors showed a substantial increase in the last 2 decades. Thus, other reasons besides reclassification should be considered to explain these unfavorable trends.

The availability of better diagnostic tools is one explanation for the increase in GIST incidence. Remarkable improvement in imaging technologies in recent years could have led to an overdiagnosis of GISTs, particularly at early stages. The National Comprehensive Cancer Network Oncology^21^ and the European Society for Medical Oncology clinical practice guidelines for GIST diagnosis have remained almost unchanged in the last 2 decades.^22^ These recommendations include endoscopic ultrasound and imaging, mainly with computerized tomography as the initial workup. Consideration of magnetic resonance imaging (MRI) is recent. Although positron emission tomography is an alternative for assessment of response or diagnosis of suspicious metastasis, it is not included in the initial workup recommendation. Thus, we do not think that the use of MRI could have greatly affected the rate of new GIST diagnosis. The recommendations for definitive diagnosis of GIST (using histology and expression of *KIT*, CD34, and *DOG1*) have also remained unchanged over time.

Another point suggesting that the increase in GIST incidence is unrelated to a diagnostic effect is the similar patterns (in strata of sex, age, primary indicator, and income) observed across most compared groups. Endoscopic procedures differ for diagnosis of GISTs in the organs studied. In this context, a potential improvement in a technique for gastric GISTs would not affect the diagnosis of small intestine GISTs. Notably, we observed increases in both early- and late-stage GISTs, suggesting that the increased incidence cannot be attributed solely to improved diagnosis. On the other hand, racial and socioeconomic disparities in endoscopy access were suggested previously,^23,24,25^ with Black and Hispanic individuals having less access than White individuals.

Mutations or silencing of the genes encoding for the succinate dehydrogenase enzyme complex^26^ or in the *NF1* gene are associated with GIST. However, the proportion of GISTs attributed to these conditions is low. Considering that reclassification and diagnostic accuracy do not seem to explain the unfavorable trends we observed, the possibility of an increase in the prevalence of a known risk factor such as obesity is plausible. Accumulating evidence suggests that GIST risk increases among individuals with obesity.^27^ We also hypothesized the existence of an unrecognized environmental or lifestyle exposure. Therefore, further etiological studies are warranted, ideally under the framework of an international research consortium.

To our knowledge, this study is the most updated SEER analysis with a detailed evaluation of race and ethnicity and organ sites. Our results are consistent with 2 previous studies using SEER data up to 2015.^19,28^ Both studies documented increased GIST incidence and found a higher burden in Black individuals compared with White individuals. Another study using data from the US Cancer Statistics database reported an overall increase in GIST incidence from 2001 to 2015 and greater rates for male individuals, Black individuals, localized disease, and the stomach as the primary tumor location.

Our survival analysis revealed divergent outcomes by race and ethnicity for the upper digestive organs. Notably, for both esophageal and gastric GISTs, Black individuals had higher mortality rates compared with White individuals. These differences could be explained by variations in patient care practices and access to health care services. Surgery is the recommended treatment for localized gastric GIST. The availability of an expert surgical team at the appropriate time is critical for favorable survival. Locally advanced and metastatic GIST is managed with targeted therapy. Tyrosine kinase inhibitors targeting *KIT* or *PDGFRA* have changed GIST survival. Imatinib (the first TKI approved by the US Food and Drug Administration in 2001^29^) is indicated in first-line treatment and in the adjuvant setting for localized resected high-risk GISTs. After imatinib, sunitinib, regorafenib, and (in recent years) avapritinib and ripretinib have been incorporated in different lines and indications in the advanced setting. Access to these high-cost agents is much more complex compared with conventional chemotherapy (with no role in GIST), particularly for patients with inadequate insurance coverage. Thus, unequal access to expensive treatments exacerbates existing disparities among disadvantaged populations.

Four SEER studies have examined overall or site-specific GIST survival (or both) from 1998 to 2018.^15,19,28,30^ Most of the studies reported that factors such as male sex, Black race, older age at diagnosis, advanced stage at diagnosis, tumor size greater than 5 cm, poorly and undifferentiated grade, and early year of diagnosis are associated with worse GIST-specific survival. Similar results were observed in our study, which included organ-specific analyses.

Molecular subtyping of GIST is an important factor for both prognosis and estimation of response to TKIs.^31^ Wild-type GIST is considered resistant to imatinib and is associated with different alterations, such as succinate dehydrogenase deficiency and neurofibromatosis type 1 or Ras–mitogen-activated protein kinase pathway alterations, among others.^32,33^

### Limitations

Our study has some limitations. First, individual-level data on socioeconomic factors and health care access are not available in the SEER datasets; thus, additional studies are needed to better understand how these factors may explain the risk and survival differences observed. Second, although the SEER registries use standardized codes and procedures for classifying race and ethnicity data, misclassification is possible. Third, data on prognosis factors are incomplete or missing. Unfortunately, the lack of information on molecular classification limits inferences on prognosis and TKI access.

## Conclusions

In this cohort study, the increasing incidence of digestive GISTs among several population groups could not fully be explained by coding reclassification and advances in diagnostic technologies. Future research should explore lifestyle-related or environmental factors underlying the unfavorable trends observed. Despite the generally good prognosis of patients diagnosed with early-stage GIST, Black individuals disproportionately die due to gastroesophageal GIST. Targeted prevention efforts are needed to decrease disparities in racial and ethnic marginalized populations.

## Notes

Supplement 1. **eTable 1.** Selected Characteristics of Patients Aged 20 Years or Older Diagnosed With Gastrointestinal Stromal Tumors, SEER-22, 2000-2019 **eTable 2.** Annual Percent Changes (APC) and 95% CIs of Gastrointestinal Stromal Tumors by Sociodemographic Variables and Racial and Ethnic Group, SEER-22, 2000-2019

Supplement 2. **eFigure 1.** Organ Site-Specific Trends* for Gastrointestinal Stromal Tumors (GIST) and Leiomyosarcomas, SEER-22, 2000-2019 **eFigure 2.** Five-Year Relative Survival (Percentage) for Gastrointestinal Stromal Tumors of Gastric and Small Intestine Origin, SEER-17, 2000-2019

Supplement 3.
Data Sharing Statement
