# Supplementary material for: Incidence and Survival Outcomes of Gastrointestinal Stromal Tumors
Source: JAMA Netw Open. 2024 Aug 19;7(8):e2428828. doi: 10.1001/jamanetworkopen.2024.28828 (PMC11333982; doi:10.1001/jamanetworkopen.2024.28828)
Supplement: Supplement 2. — eFigure 1. Organ Site-Specific Trends* for Gastrointestinal Stromal Tumors (GIST) and Leiomyosarcomas, SEER-22, 2000-2019 eFigure 2. Five-Year Relative Survival (Percentage) for Gastrointestinal Stromal Tumors of Gastric and Small Intestine Origin, SEER-17, 2000-2019 [file jamanetwopen-e2428828-s002.pdf]

Supplementary Online Content

Alvarez CS, Piazuelo MB, Fleitas-Kanonnikoff T, Ruhl J, Pérez-Fidalgo A, Camargo MC. Incidence and survival outcomes of gastrointestinal stromal tumors. *JAMA Netw Open*. 2024;7(8):e2428828. doi:10.1001/jamanetworkopen.2024.28828

eFigure 1. Organ Site-Specific Trends* for Gastrointestinal Stromal Tumors (GIST) and Leiomyosarcomas, SEER-22, 2000-2019

eFigure 2. Five-Year Relative Survival (Percentage) for Gastrointestinal Stromal Tumors of Gastric and Small Intestine Origin, SEER-17, 2000-2019

This supplementary material has been provided by the authors to give readers additional information about their work.

© 2024 Alvarez CS et al. *JAMA Network Open.*

eFigure 1. Organ Site-Specific Trends* for Gastrointestinal Stromal Tumors (GIST) and Leiomyosarcomas, SEER-22, 2000-2019

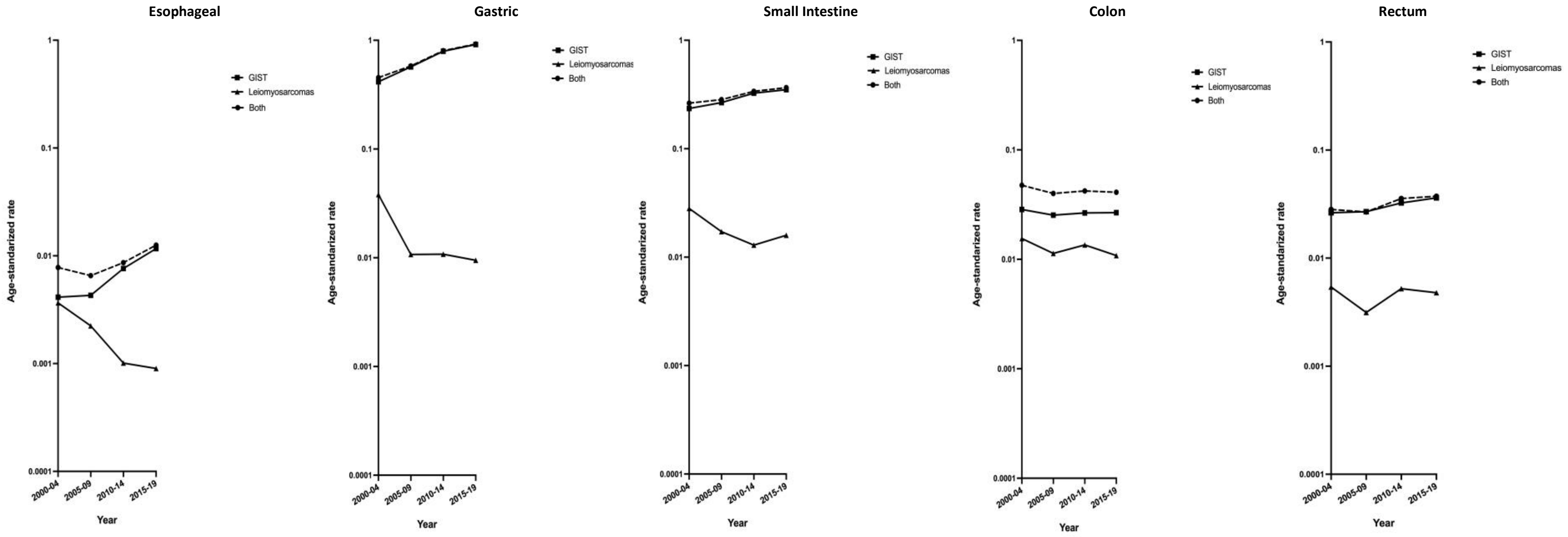

p=0.0002 p=0.06 p=0.0002 p=0.4 p=0.3

* 5-year periods were plotted to stabilize the age-adjusted rates.
p-values represent a test of differences in the trends between GIST and leiomyosarcomas by pairwise tests of parallelism (based on segmented line regression models).

© 2024 Alvarez CS et al. *JAMA Network Open.*

eFigure 2. Five-Year Relative Survival (Percentage) for Gastrointestinal Stromal Tumors of Gastric and Small Intestine Origin, SEER-17, 2000-2019

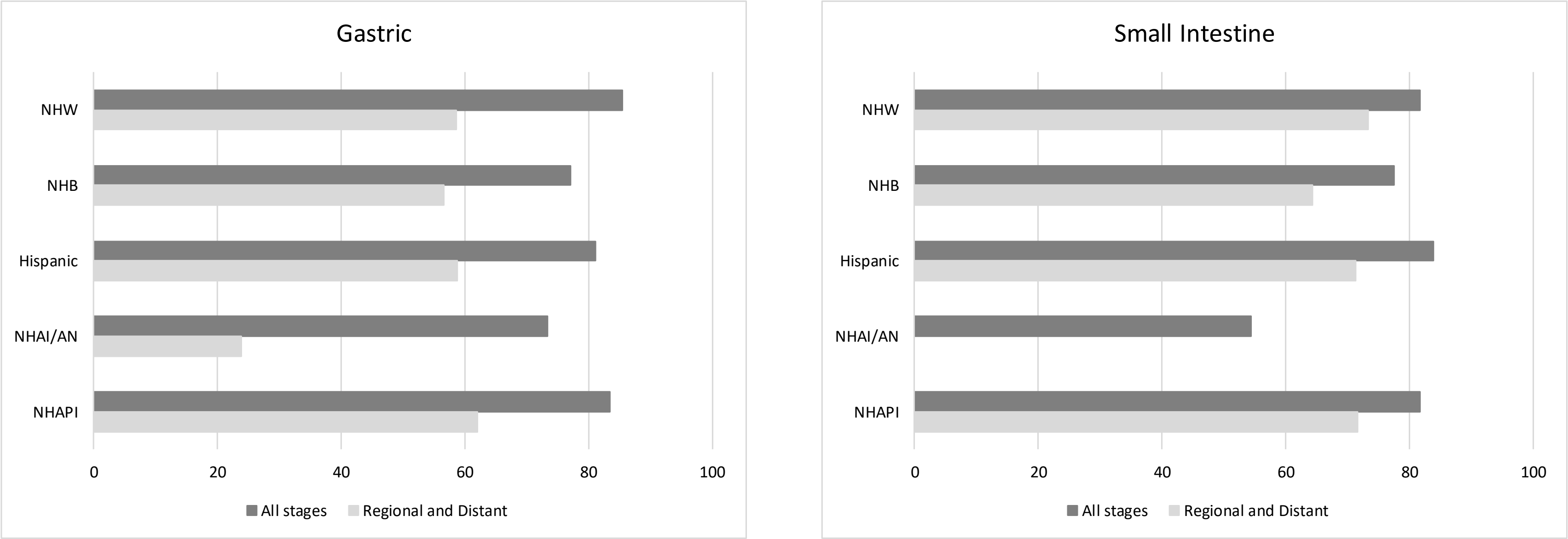

Abbreviations: NHW, Non-Hispanic White; NHB, Non-Hispanic Black; NHAI/AN, Non-Hispanic American Indian/Alaska Native; NHAPI: Non-Hispanic Asian or Pacific Islander

© 2024 Alvarez CS et al. *JAMA Network Open.*
