# Supplementary material for: Incidence and Survival Outcomes of Gastrointestinal Stromal Tumors
Source: JAMA Netw Open. 2024 Aug 19;7(8):e2428828. doi: 10.1001/jamanetworkopen.2024.28828 (PMC11333982; doi:10.1001/jamanetworkopen.2024.28828)
Supplement: Supplement 3. — Data Sharing Statement [file jamanetwopen-e2428828-s003.pdf]

Data Sharing Statement

Alvarez. Incidence and Survival Outcomes of Gastrointestinal Stromal Tumors. *JAMA Netw Open*. Published August 19, 2024. doi:10.1001/jamanetworkopen.2024.28828

Data
Data available: Yes
Data types: Other (please specify)
Additional Information: Please see below
How to access data: The data are available publicly with a data use agreement from the U.S. National Cancer Institute (https://seer.cancer.gov./data/)
When available: With publication

Supporting Documents
Document types: None

Additional Information
Who can access the data: anyone requesting the data
Types of analyses: for any purpose
Mechanisms of data availability: with a signed data access agreement
